# What older people and their relatives say is important during acute hospitalisation: a qualitative study

**DOI:** 10.1186/s12913-022-07981-9

**Published:** 2022-04-29

**Authors:** Nina Mickelson Weldingh, Marit Kirkevold

**Affiliations:** 1 Division of Research and Innovation, Department of Research Support Service, Akershus University Hospital, Lørenskog, Norway; 2 Faculty of Health Sciences, Department of Nursing and Health Promotion, Oslo Metropolitan University, Oslo, Norway

**Keywords:** Cognitive impairment, Age-friendly hospital, Patient experiences, Older people, Family involvement, Acute hospitalisation, Acute care

## Abstract

**Background:**

Due to the growing population of older people across the world, providing safe and effective care is an increasing concern. Older persons in need for hospitalisation often have, or are susceptible to develop, cognitive impairment. Hospitals need to adapt to ensure high-quality care for this vulnerable patient group. Several age-friendly frameworks and models aiming at reducing risks and complications have been promoted. However, care for older people must be based on the persons’ reported needs, and relatives are often an important part of older persons’ social support. The primary aim of this study was to explore older peoples’ and their relatives’ experiences of acute hospitalisation and determine what is important for them to experience a good hospital stay. The study was not limited to patients with cognitive impairment; but included a wider group of older individuals vulnerable to developing delirium, with or without an underlying chronic cognitive impairment.

**Methods:**

This study had a qualitative research design in which people aged 75 years or older and their relatives were interviewed during an acute hospitalisation. The study was conducted at two medical wards at a large university hospital in Norway, and included a total of 60 participants. All interviews were informed by a semi-structured interview guide and were thematically analysed.

**Results:**

Four major themes were identified in the older people’s and the relatives’ descriptions of how they experienced the hospital stay and what was important for them during the hospital stay: being seen and valued as a person, individualised care, patient-adapted communication and information, and collaboration with relatives. The themes span both positive and negative experiences, reflecting great variability in the experiences described. The presence of these four characteristics promoted positive experiences among patients and relatives, whereas the absence or negative valuation of them promoted negative experiences.

**Conclusions:**

The findings underscore the interrelatedness of older people and their relatives and that patients and relatives are quite consistent in their experiences and opinions. This suggests that listening to the concerns of relatives is important, as they can voice the older patient’s needs and concerns in situations where older people might find it difficult to do so. Furthermore, the results underscore how ‘small things’ matter in relation to how health professionals capture the patient’s individual values, need for care, information and involvement of relatives and that these are essential to ensure predictability and security and a good stay for older people and their relatives.

## Background

The number of older people in the world is growing rapidly, with an estimated doubling of persons over 60 years between 2015 and 2050 [1]. Comorbidity, polypharmacy and impaired physical and cognitive functioning are common in old age, and older people require hospitalisation more frequently. One-third of older people presenting in the hospital emergency department have some type of cognitive impairment (CI) [2, 3]. Dementia and CI are significant risk factors for developing delirium and other complications during a hospital stay [4–6]. Furthermore, older people are more vulnerable to complications and poor outcomes because of accumulating frailty and disability [7, 8].

Thus, providing safe and effective care to older people is an increasing concern, and hospitals need to adapt to ensure high-quality care for older inpatients.

To address these challenges, several models have been designed to improve the care to older people in acute hospital care [9–17]. Such ‘age-friendly hospitals’ (AFH) aim to establish systems and evidence-based practices that support high-quality care for older people. Some of the previous contributions to age-friendly hospital care, including the ‘Elder-Friendly Hospital’ programme [13], ‘The Hospital Elder Life Program’ (HELP) [13] and the ‘Acute Care for Elders’ (ACE) strategy [14], focused primarily on the recognition of risks and prevention of adverse consequences. The World Health Organization (WHO) proposed a set of age-friendly principles in 2008, seeking to optimise care for older people. These principles also took into account the older peoples’ needs and satisfaction with care. Subsequently, the Institute for Health Improvement (IHI) developed the Age-friendly Health Systems Model framework (4Ms framework), which emphasises four core elements: ”what matters” (knowing and acting upon the older person’s health outcome goals and care preferences); “medications” (adjusting medications and dosages to be age-friendly); “mobility” (individual mobility plans and suitable environments that enable mobility); and ”mentation” (adequately managing dementia, delirium and depression) [9]. Several evaluations of these models have demonstrated improvement in patient outcomes, such as quality and safety outcomes (e.g. reduced falls, delirium and pressure ulcers). System outcomes, centred around an acute care setting, including decreased length of stay, readmission and reduced direct cost of care per patient, have also been demonstrated [13, 14, 16, 18]. Less attention has been paid to whether they capture the needs of older people and their relatives.

In recent years, person-centred care (PCC) has been the recommended approach in caring for older people, particularly people with cognitive impairment [19, 20]. However, this recommendation has proven challenging to comply with in modern hospital settings with busy environments and short lengths of stay [9]. PCC emphasizes the importance of including the perspectives and preferences of the persons themselves when planning and providing care [19, 20]. So far, limited emphasis has been put on exploring both older people and their relatives’ experiences with acute hospital settings and their perspectives on what matters to them during the hospital stay. To carry out person-centered care there is a need to see the whole person in context, which may include the needs of both patients and relatives.

The current study is part of a larger study aimed at designing and evaluating a “dementia-friendly hospital program” based on previous models of age friendly care and delirium prevention models. The program was tested in a controlled clinical trial at a large university hospital in Norway in 2018-2019. The dementia-friendly hospital program consisted of an educational program, use of a screening tool of CI and delirium, and protocols for preventing and treating delirium. The primary aim of the larger study was to explore whether the “dementia-friendly” hospital program improved the detection rate of CI and/or delirium and the initiation of preventive and treatment care measures for these patients at medical wards. Results from the trial will be published elsewhere. The aim of this qualitative study was to provide insights into the experiences by older people and their relatives. This knowledge contributed to developing the model in accordance with the older people’ and relatives’ reported experiences and needs.

Previous studies of older people’ and relatives’ experiences with acute hospitalisation have described the hospital stay as a challenging time for the older people, especially for those with cognitive impairment. This is due to the narrow focus on the somatic cause of admission and insufficient emphasis on the patient’s individual needs for support and care [21–24]. Experiences reported in previous studies indicate that patients’ needs for social contact, dignity and respect are often ignored, resulting in patients feeling devalued [23, 24]. Furthermore, their experiences are influenced by the health professionals’ communication practices and degree of family involvement [25–27]. Current knowledge also indicate that relatives and informal carers often desire to be involved in the care but often experience a loss of control as they feel devalued by clinicians [22, 26–29].

While there are previous studies describing patients’ experiences with acute care, most have limited samples and have focused on patients with dementia or subgroups of dementia. In this study, the focus was not limited to the patients diagnosed with dementia, but included a wider group of older individuals vulnerable to acute CI or delirium, with or without an underlying chronic CI. This broader group of persons represent a vulnerable patient group frequently encountered in acute care settings. Although naming the model dementia-friendly, we assumed that the model of care should address the needs of this wider group in high risk of developing acute CI (delirium) during the hospital stay.

At the time when this study was carried out, there were few studies available capturing both patients’ and relatives’ experiences of the same hospital stay. We believe this study contributes new insights by shedding light on how the experiences of frail older people and those of their relatives are linked and influence each other and that looking at these interlinked experiences can support a more holistic care model for vulnerable older people during an acute hospital stay. Furthermore, the study setting is non-geriatric medical wards, which is important as older people are often admitted to wards related to their acute somatic cause, rather than to wards with expertise on geriatric patient care. This knowledge may guide health professionals and policymakers in designing health services that facilitate more age-friendly care for acutely hospitalised older people in general medical wards.

### The aim of the study

This study aimed to explore how older people and their relatives experience acute hospitalisation and what they emphasise as important for them during the hospital stay.

## Methods

### Design

This study had a qualitative research design, using individual interviews with older hospitalised people and their relatives. A qualitative methodology is well-suited for exploring people’s individual views and experiences while considering the descriptions as part of a social context, such as an acute care setting [30].

### Setting and participants

This sample was recruited from a larger controlled clinical trial, evaluating the impact of a dementia-friendly program in acute-care hospital units. The study took place in two medical wards (a pulmonary and a cardiac ward) at a large acute-care hospital in Norway with a catchment area of 600,000 inhabitants. The decision to use a pulmonary and a cardiac ward was made based on the aim of including older people`s experiences of an acute hospital stay in medical wards without particular focus of the geriatric patient.

Potential participants in the controlled clinical trial were patients 75 years of age or older who were admitted to one of the wards for acute medical illness between October 2018 and December 2019. Exclusion criteria included critical illness, inability to communicate (whether from aphasia, severe hearing loss, or inability to speak Norwegian) or isolated because of severe infection. Potential participants in this qualitative study had been screened for CI and delirium using the 4 ‘A’s Test (4AT). 4AT is a brief orientation measure which includes cognitive screening sensitive to general CI, in addition to items on altered level of alertness and change in mental status, which are strong indicators of delirium [31]. A 4AT score of four or more indicates delirium, while a score of 1–3 indicates CI [32]. Patients who had a ‘positive 4AT score’ (a score of 1 or more) and thus an indication of CI, either at admission or during the hospital stay, were potential participants in an interview. Notably, 4AT is not a diagnostic tool indicating that we do not know whether all patients with a positive 4AT score had a CI. Furthermore, long-term cognitive impairment, such as dementia, needs extensive diagnostic tests and should not be diagnosed during an acute hospitalisation. Nevertheless, the 4AT score gave us an indication of cognitive function and an opportunity to include older people with different degrees of cognition, thereby capturing a broader range of experiences and needs of older hospitalised people and relatives.

Consent competence was assessed based on the 4AT screening and a clinical assessment by clinical research nurses. Patients who where cognitively able to understand the study and had sufficient physical health to participate in an interview were invited to participate by a clinical research nurse and signed a written consent form. In cases with a high 4AT score and suspicion of delirium, relatives were consulted and asked to give consent in addition to the patient, or a new 4AT score was performed before interview invitation to make sure the patient was not delirious at the time of interview and had sufficient cognitive capacity to consent.

Patients were invited to participate face to face by being visited in the ward and relatives were recruited either face to face when visiting the patient or through a telephone call. The patients decided whether they wanted a relative present during their interview. As we aimed to capture the range of experiences and views among older people during an acute hospital stay, a purpose-full sampling was done, where we sought to include participants who varied in terms of age, gender, severity of cognitive impairment and relation to their relative (spouse, parent, etc.). A total of 60 participants participated in the interviews, (33 patients and 27 relatives). Sample characteristics are presented in Table 1. Even though twelve of the participating patients had a negative 4AT score (4AT=0) at admission, these patients had a positive 4AT score (4AT>4) taken during the hospital stay.

**Table 1:** Characteristics of the sample

Characteristics			*N=60*
Informant interviewed n (%)	Patients 33 (55.0)	Relatives 27 (45.0)	Total 60 (100)
Gender			
Female, N (%)	14 (23.3)	21 (35.0)	35 (58.3)
Male, n (%)	19 (31.6)	6 (10.0)	25 (41.6)
Age, years min-max (median)	75-94 (84.4)		
Admission ward			
Cardiac, n (%)	18 (54%)		
Pulmonary, n (%)	15 (45%)		
Somatic cause of admission			
Heart failure, n (%)	11 (33%)		
Pulmonary disease, n (%)	10 (30%)		
Endocrinologic disease, n (%)	2 (6%)		
Infection, n (%)	3 (9%)		
Acute dysfunction, n (%)	7 (21%)		
Cognitive function (4AT score) at admission, cat.			
No suspicion of cognitive impairment (4AT=0), n (%)	12 (36%)		
Suspicion of cognitive impairment (4AT=1-3), n (%)	18 (55%)		
Suspicion of cognitive impairment or delirium (4AT≥4), n (%)	3 (9%)		
Length of hospital stay, days mean (min-max, median, mode)	9 (2-28, 6, 4)		
Relation of relative			
Husband/Wife, n (%)		9 (33.0)	
Daughter, n (%)		12 (44.0)	
Son, n (%)		5 (18.5)	
Niece/nephew, n (%)		1 (0.3)	

### Data collection

Data were obtained using individual semi-structured interviews of patients and relatives. Each participant was interviewed once during the hospital stay for 20–90 minutes. The interviews focused on their immediate experiences with the hospital stay. Previous research [23] has shown that people with cognitive impairment can express their own experiences, views, thoughts and feelings related to the "here-and-now" situation. The participants were also asked to give their suggestions on how to improve the hospital stay. The interviews were conducted based on a thematic interview guide and addressed the patient’s and relative`s experiences related to the hospital stay, including both positive and negative experiences. Examples of questions were: Please tell us about your experiences being here at the hospital/your relative being here at the hospital. How do you experience being welcomed by the health professionals? What are your thoughts concerning the treatment you/your relative receive? Follow-up questions were asked based on what the participants answered. They were also asked to give their suggestions on how the hospital stay could have been improved to provide adequate care for patients such as themselves. All interviews with the patients took place on the ward as a face to face interview, either in the patient’s room or in a separate room in cases where the patient shared room with another patient. Interviews with relatives were performed either face to face in a separate room at the hospital or by telephone. Interviews with the relatives were conducted close to discharge from the ward, or as a telephone interview shortly after discharge. All interviews were recorded using a tape recorder and transcribed verbatim by Mrs. NMW. Field notes were taken during the interviews for reference during coding. The study was conducted by two female researchers with nursing backgrounds and research training at the master’s and PhD levels. One has extensive experience with qualitative methods and research on geriatric nursing care (MK), while the other has extensive experience in the acute care of older people (NMW).

### Data analysis

All interviews were transcribed by NMW. All data were then analysed using the six-phase approach to thematic analysis described by Braun and Clarke [33–35]. This is a method for identifying, analysing and reporting patterns in qualitative data, and the approach has been widely used and accepted as robust across a wide range of disciplines. In the first phase, both authors (NMW and MK) read through the entire dataset to get an initial understanding of the data. NMW then reviewed all data and developed codes that captured the patients’ and their relatives’ positive and negative experiences related to the hospital stay. These codes were reviewed by co-author MK to ensure agreement between the codes and the data. Initial differences were discussed until a consensus was reached. An inductive approach was followed in which NMW and MK independently reviewed the data index for each code and then categorised the codes into potential themes. The themes were compared and discussed in terms of relevance to the research questions until a consensus was achieved. The themes were then refined to ensure that each initial theme was distinct from the other themes, while the data within the themes cohered together meaningfully. Finally, the titles of the themes were discussed back and forth to be as concise as possible. We have used the consolidated criteria for reporting qualitative studies (COREQ) [36] when reporting this study.

### Ethical considerations

Ethical approval for the study was obtained from the local officer for data protection and the Regional Committee for Medical and Health Research Ethics in Norway (2018/666). All patients were asked to give written consent for the use of data collected in this project.

## Results

**Fig. 1:**
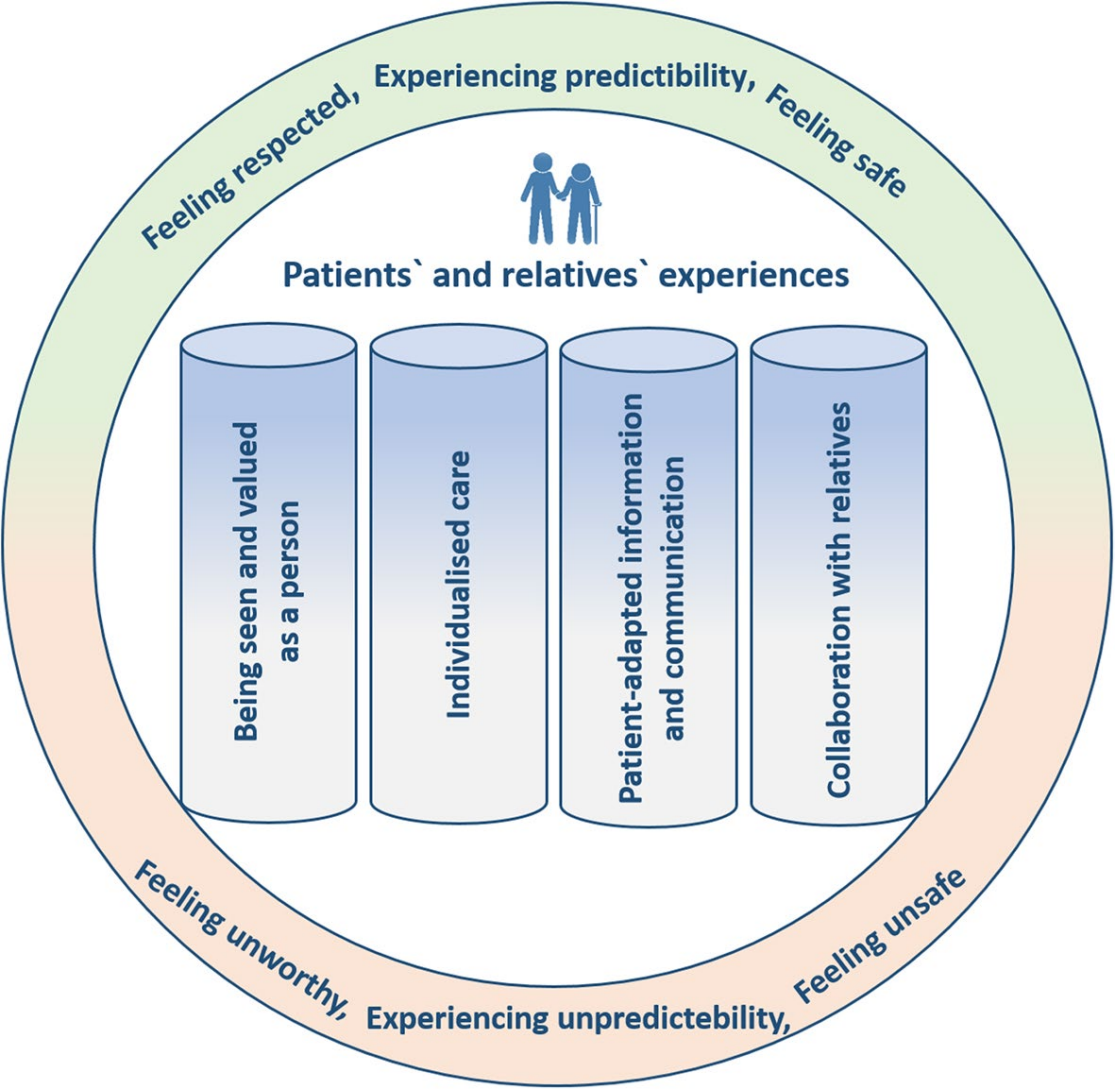
Important aspects of patients’ and relatives’ experiences of acute care hospital stay

We identified four major themes (Fig. 1) that capture what the patients and their relatives experienced as important for them during the acute hospital stay: being seen and valued as a person, individualised care, patient-adapted communication and information, and collaboration with relatives. The themes span both positive and negative experiences, reflecting great variability in the experiences described. The material can be understood as a continuum between the two opposites of positive and negative experiences. Patients and relatives describing these four positive experiences reported a positive hospital stay, whereas the opposite, absence or negative valuation of these themes, promoted negative experiences of the hospital stay. Patients and relatives reported similar experiences. When describing a positive hospital stay, patients experienced being met and respected for who they were and their own needs, which contributed to a feeling of predictability and safety for both patients and relatives. If, on the other hand, these were not present, the hospital stay was experienced as unpredictable and, for some, unsafe. The negatively ‘coloured’ experiences contributed to patients and relatives experiencing not having their needs met or feeling unworthy, which led to disappointment and even resentment. In the following, the four themes are delineated in further detail.

### Being seen and valued as a person

The patients’ and relatives’ experiences emphasised the importance of each patient being seen and recognised as a person who is valued and met with respect, honesty, kindness and compassion, without the feeling of being objectified and treated as a ‘parcel on an assembly line’, as one patient described it. Another patient with positive experiences related to this described it as a form of sincere kindness:

*‘It is the kindness and … I want to emphasise, the sincere kindness. There is a difference… Kindness can be acquired quite easily, but the honest kindness is the one you see in people [here]’.* Patient (P)1

Several aspects of the hospital stay influenced the experiences, colouring them as primarily negative or positive. The relatives having positive experiences during the hospitalisation valued that the health personnel communicated directly to the patients, as this contributed to each patient feeling respected as a person. There was also a recognition that ‘being seen and valued as a person’ was closely connected to how much time the health professionals spent with the patient. Both patients and relatives describing positive experiences found it reassuring that the health professionals visited the patient often, often outside of routine tasks, and spent time listening to what the patient had to say, even though they were in a hurry and had many other patients to look after:

*‘I have not seen anything like it. I have been so safe, I have not been anxious for an hour, they have been here so often’.* P2

*‘In a way it is always like: “Here is the leash!”—coming by and [saying]: “Just pull this, I will come again”. And if she does not pull the bell, they will come by anyway. I think it is absolutely fantastic how she has been followed up; an incredible number of skilled people work there’*. Relative (R)1

These patients and relatives generally expressed great trust in, and respect for, the health professionals for doing a great job despite limited available time. Even though they recognised the constant bustle in the hospital ward, they felt safe and taken care of. The following quotes illustrate the sentiments among these participants:

*‘They run several kilometres a day and are doing the best they can. You cannot ask for more’.* P3

*‘I think they are completely unique. Ring this bell and they will come right away… if they are not somewhere else. Sure, they cannot be everywhere at once’.* P4

Furthermore, being taken seriously and listened to were closely linked to positive experiences. Both patients and relatives expressed the importance of being closely followed up and assured by the doctor that they were doing everything they could for the patient and were not giving up on them just because they were old:

*‘It is important…that she is taken seriously, even though she is almost 90 years old….I think that is important. You are not getting scooped up in a corner because you are too old, and I think that is very good.’* R2

In contrast to the positive experiences of being seen and valued, this sample also reflects negative experiences in relation to feeling seen and valued as a person. Several patients and relatives felt that the care was not consistent. Their experiences varied greatly depending on the individual health professional who cared for them. They described large differences in helpfulness and understanding among the health professionals. Some of the patients expressed that when you are a vulnerable patient unable to speak up for yourself, you are at the mercy of the person you meet, which ought not to be the case:

*‘When you wear this gown, you are put together with all the others. They do not greet one, and there are many who… they just shuffle past and look straight ahead’.* P5

Many patients expressed feeling lonely and bored during the hospital stay, and that their psychological and social needs were often not met. Some patients would have loved to take a walk outside the patient room, either to talk to other co-patients or just to see something other than the walls inside the patient room. However, they experienced that no one saw them or their needs; the health professionals was busy and concerned with medical issues and task-oriented care. If anyone happened to come into their room, it was just because they had some routine tasks to accomplish. This led to doubts about whether the health professionals was able to provide adequate care at all:

*‘I think both me and my mother experience it as an assembly line. There is no care in those who work there. You are just a number in the line, and everything goes on autopilot and then out…done with you’.* R3

Furthermore, when patients experienced being left alone in the patient room and felt that no one had time to look after them, they often felt totally ignored and unworthy:

*‘They just drive you into a “hole”, and then there you go… just lie here until we have time for you’.* P6

One patient expressed that she thought the health professionals were tired of her; when she called the bell, she was told that she had to wait because they had many others to take care of. This left the patient with feelings of anger, being a burden and greater vulnerability:

*‘I know there are others here….but when I’m in pain, I need help…’*. P6

### Individualised care

The patients and the relatives emphasised that one of the most important aspects contributing to a positive hospital stay was receiving help with their individual problems and knowing that something was being done and that they experienced getting better:

*‘I have had such a good time during my hospital stay, I could not wish for more. The nurses, doctors, everyone … they got me on my feet’*. P2

Relatives appreciated when the health professionals were well prepared and well informed about patients’ care needs. This was especially important for patients with cognitive impairment. The relatives expressed satisfaction with the health professionals’ focus on the patients’ wellbeing and creating a calming and less stressed environment so the patient felt safe and calm even though they were in an unfamiliar environment. As one relative explained:

*‘I think a lot is good…. they know that my dad has a hearing aid in his right ear, they know that they have to speak loud and clear. They know they have to look at him when they talk to him. And they ask questions back to him, to hear if he has understood correctly. In other words, they have read the patient journal and they know what they are supposed to do in relation to communication and how the patient is doing’.* R4

Some relatives experienced that the care focused primarily on the diagnosis of relevance for the unit in which the patient was admitted, rather than the person as a whole human being. These individuals experienced that the health professionals had special competence in their particular fields (e.g., heart or lung diseases), but not the competence to address other matters, which is necessary in relation to care of older people. For example, patients and relatives articulated that sometimes patients with cognitive impairment have problems communicating their own needs and that they often misunderstand things. Thus, to help these patients with their individual needs, the health personnel must have knowledge on how to adapt care to patients with cognitive impairment:

*‘You have to try to treat each patient differently based on each diagnosis, and if there are several diagnoses, they must try to see the whole… like how can we try to do the best for this patient? But it is not so easy to do something for a patient who is not quite able to say what he wants’.* R5

Relatives articulated that the health professionals was not aware of how serious the patient’s cognitive impairment and memory loss actually was and the serious consequences it could lead to if the health care personnel were not careful enough. Sometimes the patients can say no to a question when they actually mean yes—or they may know what they want to tell, but they do not remember the words and do not manage to express it. The patients expressed that they sometimes felt stupid and did not always manage to think clearly. One patient explained that his tongue did not always cooperate and that it was difficult to express himself. Relatives of patients with cognitive impairment often experienced that the patients did not question anything, and sometimes they did not manage to ask for painkillers. Thus, they could be suffering from pain for a long period if the health professionals did not help them to convey their needs:

*‘Even though you have dementia, and may not manage to express yourself, you still feel pain’.* R6

Furthermore, patients with cognitive difficulties often forgot to ring the bell when they needed help or forgot that they could not walk, thereby needing supervision more often than other patients.

### Patient-adapted information and communication

There was some variation in how patients and relatives experienced receiving the information they needed. Positive experiences of the hospital stay were connected to relatives and patients having adequate and patient-adapted information during the hospital stay. The patients wanted the health professionals to inform them before procedures and about further plans regarding discharge. Thus, they did not need to ask a lot of questions and felt safe and assured that things were being taken care of:

*‘Patient: I get to know what I need without digging and asking...It has been sufficient. And then, when I have got that information, I do not need to bother, or fuss any further about the discharge’.* P7

Although some experienced having their information needs covered, the experiences in relation to information were mostly negative. Several patients and relatives articulated that they did not receive any information if they did not ask for it. Some also experienced difficulties getting relevant information because they did not know what to ask for:

*‘Well, if I was going to get any information over there, I at last had to ask. I didn’t get any information without asking’.* R7

*‘Sometimes they say, “Ask over there,” and then there are two of the health professionals talking to each other and then you don`t quite know if you can disturb while they are talking… so if they could just come to us and inform us a little…’.* R8

Furthermore, patients also asked for more information about what the plan for the day was instead of having to sit and wait for the doctor to come. Such a lack of timely information made patients feel very confused and impatient:

*‘It would have been much easier to be here if I had known, instead of sitting here and just waiting…When will someone come and what’s next?… It seems like it’s all so secret’.* P5

Most patients and relatives articulated that it was extremely difficult to get in contact and get information from the doctors because they were always in such a hurry. The relatives frequently experienced having to trust the patient to get informed, which was especially difficult in cases where cognitive impairment was present, and the relatives worried that the patient had not comprehended the information provided:

*‘It frustrates me more than anything else, not getting answers…not getting in touch with the doctors. I know they have a lot to do, but it’s frustrating as a relative. Especially if you have a patient with cognitive impairment’.* R9

These experiences of extensive waiting and few answers were often related to experiences of unpredictability for both patients and relatives.

For the patient to receive adequate information, the health professionals also had to be aware of the patient’s prerequisites for processing information. Even though many patients experienced getting information, they also conveyed that they could not always remember what they had been told. Relatives expressed that the patient received too much information, and they often did not remember any of it and ended up believing that something completely different was wrong with them. The relatives also highlighted the value of having one responsible doctor, which they described as highly important for patients with cognitive impairment.

*‘I experience that the treatment becomes so diffuse, that one doctor decides one thing and then another doctor comes and he decides something else, and then a third doctor decides a third thing. You see, there are too many “cooks”, and my dad is getting completely confused, he does not understand anything of it’.* R4

The patients also desired involvement in decision-making regarding treatment and discharge, including recognition of their needs. They expressed that early discharge planning provided predictability and promoted hope. However, patients and relatives often experienced inadequate preparation for discharge and a lack of information from the health professionals, resulting in experiences of inappropriate care and worries about the time after discharge. This also often led to the impression that the patient was being discharged before finishing their treatment:

*‘I have an impression that the pressure to get a free bed is so great that when there suddenly were many beds in the corridor, he had to leave the hospital, and then I only get a phone call that he had to be picked up in an hour’.* R10

### Collaboration with relatives

Both patients and relatives highlighted the importance of involving the relatives throughout the hospital stay. The relatives experienced responsibility for the patients both during the stay and after discharge. However, experiences varied in terms of feeling sufficiently involved in the care of the patient. The relatives explained that the patients depended on them to help them convey their needs. Relatives who were satisfied with the hospital stay expressed that they were included when the patient received information about treatment and discharge planning. They described that they could come to visit whenever they wanted and felt that their knowledge about caring for the patient was valued and considered an asset. In contrast, other relatives felt that they were not heard or taken seriously when they tried to convey the patient’s needs. Some relatives almost felt guilty as they felt that the personnel communicated that the relative had the patient admitted for no reason. Furthermore, some relatives also experienced visiting the patient as problematic and did not feel welcome outside the visiting hours:

*‘They were so set on the rules of visiting time …they were a bit brusque. He received good care, and I will not complain about that, but somehow when we understood that he would not make it, we found it a bit painful not to get to visit him if we came a little outside visiting hours’.* R11

In particular, relatives of patients with cognitive impairment often perceived themselves as key actors in terms of ensuring that the older person received care in line with his or her needs, but they sometimes experienced that their concerns were not taken seriously:

*‘They would have had to put a person on him “as a stamp” if I had not been allowed to be there. But … many [of the staff] do not understand. They probably really look at me as someone who wants to get in the way’.* R12

Several relatives were worried about leaving the patient alone in the hospital because they perceived that the patient could not speak for him/herself. They doubted that the health professionals would understand and have enough time to give appropriate care to the patient. The relatives felt that they had to take responsibility so that the patient got the care s/he needed:

*‘We experience that …… at least some of us must be there all the time and make sure that he gets what he should get and that he gets the right medication and that he gets it at the right time’.* R13

As described in the previous theme, relatives wanted to be involved when information was given and not just read the discharge papers afterwards. One relative suggested a note to relatives about what had been done with the patient during the hospital stay, the diagnosis and how they were expected to follow up after discharge. Both relatives and patients experienced this as reassuring, as the patients themselves often felt unable to perceive everything that was said and were afraid of forgetting things. The relatives highlighted the importance of being involved in the discharge planning process and being assured that the patients’ basic needs would also be taken care of after discharge, e.g., by communicating with home care services. However, they often experienced poor or insufficient communication and involvement at discharge, sometimes resulting in negative consequences when the patient returned home. Several relatives perceived that older patients often do not want to be dependent on or a burden for others, thereby saying no when asked if they need help at home. The relatives emphasised that health personnel should verify with the relatives if what the patient was saying about managing at home actually fits the reality:

*‘They had asked Dad if he needed any help when he got home, but Dad said: “No, no, it’s fine, because my daughter does not live very far away!” Things that he knows I somehow do not have the opportunity to do. And then the nurses just rely on this. Well, in this case, this was not true, so it was problematic when he returned home. They should have called and asked if it was true; your father says that when he comes home it’s easy for you to help him in the morning and just take time off from work… is that part of reality?’.* R14

## Discussion

By interviewing acutely hospitalised older people and relatives about their experiences and what they highlighted as important for them during the hospital stay, we found that experiences of security, being respected and experiencing predictability fostered positive experiences. The stories of the patients and relatives tell us that these experiences are related to four main dimensions: 1) being seen and valued as a person, 2) individualised care, 3) patient-adapted communication and information and 4) collaboration with relatives.

Few previous studies have looked at both patients’ and relatives’ experiences of a specific hospital stay. Comparing their experiences, we see that their perspectives on what is important for a good hospital stay are very similar. A prominent feature in this material is the variation between the positive and negative experiences—from those who were very satisfied and experienced being respected, receiving adequate information and having involvement of relatives to those who experienced dissatisfaction through experiencing the opposite. The experiences were located along a continuum between these extremes. Exploring the reported experiences in further detail, we see that they are not primarily about the lack of care but more about the way care is conducted. Three overall factors may contribute to explain the experiences: a) the way health professionals communicate, b) the competence of the health professionals and c) the health professionals’ working conditions. In the following, these will be further discussed in relation to the main findings. First, there was high consistency between the patients’ and relatives’ stories with regard to the importance of being met with respect and not being objectified. Several respondents pointed out the large variation in how one felt seen, heard and respected by the individual health worker, and that the communication often was task-oriented or diagnosis-focused rather than holistic and individualised. A multisite study by Godfrey et al. [32] described various aspects of patient communication that may influence the patient’s experience of respect and dignity. According to Godfrey et al. [32], health professionals often must prioritise medical and other important tasks. However, the way they communicate with the patient when carrying out the tasks is decisive for how the patient experiences being seen, heard and respected as an individual. As Godfrey et al. [37] advocate, the content of the communication may well be task-oriented, but the form may still be warm and respectful. Through that, the health professionals engage with the patient and communicate at an emotional level. Not only does this help a patient hold on to his or her sense of self; it may also provide trust between the health professionals and patients and allow for individualised care [38]. To provide individualised care, it has been highlighted that health professionals must gain knowledge of the patients’ normal health condition, including their physical, psychological, cognitive and social functioning and their interests and values, and use this in the communication and care [37, 38]. Such holistic care has been a key aspect of PCC [39], which is considered ideal and necessary in the care for older patients with cognitive impairment [40, 41]. However, most research on the implementation of PCC has focused on long-term care [42, 43], and there is no consensus regarding the important features of PCC in other contexts [44]. Delivering PCC in acute care settings has been shown to be challenging because of an often task-focused approach, busy environments and fast-paced working conditions [45].

Getting to know the person in the sense PCC requires may be difficult in an acute care setting, and Yevchak et al. [38] emphasised the need to talk with family members to gain such knowledge. In this study, some relatives reported disappointment with the health professionals for not seeking their opinion about the care of the patient. The relatives often considered themselves to be experts in the care needs of their relatives and therefore wanted to be involved in the planning to be assured that the patient would get the care he/she needed. This is in line with other studies in smaller samples and subgroups of older patients and family members, confirming that health professionals also need to consider the needs of the relative as the informal carer and the dynamics of the relationship between the patient and the relative to individualise the patient’s care [22, 46–48]. Another reason for patients and relatives to emphasise family involvement might be that older patients often authorise family members to act and participate on their behalf [47]. However, the findings from this study underscore that older people need to be seen and heard and that the relatives cannot substitute the older person`s voice. Rather, we have to see and treat the patient and the relatives as interrelated and in their wider context in order to ensure high quality PCC.

In addition to the importance of emotional communication, results from this study also highlight the need for providing factual information, understood as information about treatment and diagnosis, routines for the day and further plans after discharge. The findings also show that for the factual information to be patient-adapted, there is also a need to gain knowledge of the patients’ habitual state regarding cognitive or sensory impairment as well as information on what matters for the patient and how the family may be involved to the benefit of the patient [48].

However, gaining knowledge of the patient’s habitual state and situation outside the hospital and providing adapted communication and information may be difficult, as health professionals in acute care settings report being too busy and lacking time to provide adequate care [38]. Health professionals describe time pressure as ubiquitous in the daily care of older persons, leaving them with a sense of failure in terms of providing care [49]. This means that the reported variation in how the patients and relatives are being met by the health professionals cannot be explained by looking only at the personal characteristics of the individual health professional. The findings must also be interpreted in light of the working conditions that health professionals often describe as physically hard, emotionally demanding and stressful with competing priorities [37, 49]. Thus, the respondents’ frustration at not being seen and heard may, in part, be explained by the reported bustle and hurry in acute medical wards [37], which leaves psychosocial care for patients as a secondary concern [50]. Thus, the reported variations between positive and negative experiences may also be indirectly affected by the health professionals’ working conditions. This may also indicate a need for changing the clinical mindset and developing ways to integrate recognition of the patient in the hospital routine practice and to ensure that health professionals in acute care hospitals has the necessary resources to provide the comprehensive care that older people need [51]. Moore et al. [52] advocate that strong leadership and adaptive strategies are important for overcoming existing practices and adapting to traditional hospital care to PCC. Findings by Alhalal et al. [53] also show that lower burnout, higher compassion, satisfaction and structural empowerment increase nurses’ provision of PCC.

The variations between positive and negative experiences may also be influenced by the ward culture and knowledge about care for older people in general. It has been reported that care for patients may be compromised due to limited knowledge among acute hospital health professionals about frail older people generally and risk factors contributing to development or deterioration of cognitive impairment in particular [54]. Good care for older people requires comprehensive competence [55]. This may include advanced practice nurses with special training in geriatric care who can conduct comprehensive assessments and plan and support the care of older people with complex needs. As Henni et al. [56] advocate, organisational adjustments are needed for advanced geriatric nurses to utilise their knowledge and skills to their full potential.

### Implications for clinical practice

The study has contributed to a greater understanding and awareness of how older people and relatives experience an acute hospital stay. The finding that patients and relatives are quite consistent in their experiences and opinions is an important new insight from this study and suggests that listening to the concerns of family members is important as they can voice the older patient’s needs and concerns in situations where older people might find it difficult to do so. The study also contributes important learning points for acute medical wards, suggesting that small but important organisational adjustments can ensure that each older patient feels seen, safe, respected and valued as a person. For one, the wards need to be organised in a way that gives health professionals an opportunity to use more of their clinical expertise in the complex care for older people. This includes having sufficient time to collect baseline information about the patient in order to adjust their communication and information in relation to various sensory impairments and cognitive impairments. In addition, they must take into account that older people often need more time to both express their thoughts and receive information. Our study also highlights the importance of involving the relatives in the care for the older people. Although the relatives should not replace the older patient’s voice, relatives frequently see needs and concerns that patients and health professionals do not see, which can contribute to important information in the care for the patient. Furthermore, clinics responsible for the care of frail older people should consider measures to ensure that the health professionals have sufficient time set aside for this vulnerable group, e.g. by recognizing that a higher patient-to-health professional ratio might be necessary. Furthermore, it is the responsibility of the hospital leaders to create a culture where both the underlying values ​​and resource allocation are focused on person-centred communication and care for both patients and relatives. In this way reaching the goal of developing aged-friendly hospitals may be possible.

### Strengths and limitations

The results of this study must be interpreted in light of the relatively short interviews with the informants; thus, we have not received in-depth information in all the interviews. Furthermore, we only captured the perspectives of patients and relatives, but not the health professionals. On the other hand, 58 interviews with both patients and relatives is a large sample and captures a broad variety of experiences. The patient interviews were conducted during the hospital stay and not retrospectively, which is a strength that may have allowed us to capture many experiences and feelings that might later have been forgotten. Even though the sample in this study represents only two medical wards at one hospital, it is a large university hospital with a large admission area. Some of the interviews were performed by the two authors jointly, which provides a common understanding of the interviews. Furthermore, through the analyses, both authors reviewed the data in several rounds to ensure that we agreed on the interpretation of the content. The researchers’ different backgrounds—NMW’s experiences with clinical practice and MK’s experiences with qualitative research methods—have hopefully strengthened the design of the study by bringing supplementary perspectives to the topic at hand and by challenging and/or corroborating each other’s interpretations.

## Conclusion

This study strengthens the findings from previous studies that older people need to feel respected and that family wants to be more involved in the patient care. Furthermore, the findings underscore the interrelatedness of older people and their relatives. Hence, they must be seen in context and not isolated from each other. The older people and relatives in this study identified four themes contributing to their experiences of predictability, safety and respect, which were important to experience a good hospital stay. The results underscore how small things matter in relation to how we meet and communicate with patients and relatives, listen, get to know their individual values, needs for care and information, and best involve their relatives as the resources they are and want to be. In addition, this study shows that positive experiences are not primarily dependent on the care delivered, but more about the way care is conducted. For the staff at acute hospital wards to have the opportunities to provide the care needed by older people, organisational adjustments of working conditions may be needed.

## Data Availability

The dataset generated and analysed during the current study is not publicly available due to issues of anonymity and consent in this vulnerable population. A summary is available from the corresponding author on reasonable request.

## Abbreviations

ACE: Acute care for elders
CI: Cognitive impairment
PCC: Person-centred care
